# Trends of caesarean delivery from 2008 to 2017, Mexico

**DOI:** 10.2471/BLT.18.224303

**Published:** 2019-04-30

**Authors:** Tarsicio Uribe-Leitz, Alejandra Barrero-Castillero, Arturo Cervantes-Trejo, Jose Manuel Santos, Alberto de la Rosa-Rabago, Stuart R Lipsitz, Maria Antonia Basavilvazo-Rodriguez, Neel Shah, Rose L Molina

**Affiliations:** a Center for Surgery and Public Health, Department of Surgery, Brigham & Women’s Hospital, One Brigham Circle, 1620 Tremont Street, Suite 4-020, Boston, MA 02120, United States of America (USA).; b Division of Newborn Medicine, Children’s Hospital Boston, Boston, USA.; c Facultad de Ciencias de la Salud, Universidad Anáhuac, Huixquilucan, Estado de México, Mexico.; d Escuela de Medicina Ignacio A Santos, Tecnológico de Monterrey, Monterrey, Mexico.; e Clínica Integral de la Mujer, Gustavo Madero, México City, Mexico.; f Department of Obstetrics and Gynecology, Beth Israel Deaconess Medical Center, Harvard Medical School, Boston, USA.

## Abstract

Caesarean delivery rates in Mexico are among the highest in the world. Given heightened public and professional awareness of this problem and the updated 2014 national guidelines to reduce the frequency of caesarean delivery, we analysed trends in caesarean delivery by type of facility in Mexico from 2008 to 2017. We obtained birth-certificate data from the Mexican General Directorate for Health Information and grouped the total number of vaginal and caesarean deliveries into five categories of facility: health-ministry hospitals; private hospitals; government employment-based insurance hospitals; military hospitals; and other facilities. Delivery rates were calculated for each category nationally and for each state. On average, 2 114 630 (95% confidence interval, CI: 2 061 487–2 167 773) live births occurred nationally each year between 2008 and 2017. Of these births, 53.5% (1 130 570; 95% CI: 1 108 068–1 153 072) were vaginal deliveries, and 45.3% (957 105; 95% CI: 922 936–991 274) were caesarean deliveries, with little variation over time. During the study period, the number of live births increased by 4.4% (from 1 978 380 to 2 064 507). The vaginal delivery rate decreased from 54.8% (1 083 331/1 978 380) to 52.9% (1 091 958/2 064 507), giving a relative percentage decrease in the rate of 3.5%. The caesarean delivery rate increased from 43.9% (869 018/1 978 380) to 45.5% (940 206/2 064 507), giving a relative percentage increase in the rate of 3.7%. The biggest change in delivery rates was in private-sector hospitals. Since 2014, rates of caesarean delivery have fallen slightly in all sectors, but they remain high at 45.5%. Policies with appropriate interventions are needed to reduce the caesarean delivery rate in Mexico, particularly in private-sector hospitals.

## Résumé

Les taux d'accouchements par césarienne au Mexique sont parmi les plus élevés au monde. Au vu de la sensibilisation accrue de la population et des professionnels à ce problème et de la mise à jour des directives nationales de 2014 visant à diminuer la fréquence des accouchements par césarienne, nous avons analysé l'évolution des accouchements par césarienne selon le type d'établissement entre 2008 et 2017 au Mexique. Nous avons obtenu des données issues d'actes de naissance auprès de la Direction générale mexicaine des informations sur la santé et regroupé le nombre total d'accouchements par voie basse et par césarienne en cinq catégories d'établissement: hôpitaux relevant du ministère de la Santé, hôpitaux publics, hôpitaux relevant de l'assurance liée à l'emploi public, hôpitaux militaires et autres établissements. Les taux d'accouchements ont été calculés pour chaque catégorie à l'échelle nationale et pour chaque État. En moyenne, 2 114 630 (intervalle de confiance, IC, à 95%: 2 061 487-2 167 773) naissances vivantes ont eu lieu chaque année entre 2008 et 2017 à l'échelle nationale. Parmi ces naissances, 53,5% (1 130 570; IC à 95%: 1 108 068-1 153 072) étaient des accouchements par voie basse, et 45,3% (957 105; IC à 95%: 922 936-991 274) étaient des accouchements par césarienne, avec peu de variations dans le temps. Au cours de la période étudiée, le nombre de naissances vivantes a augmenté de 4,4% (de 1 978 380 à 2 064 507). Le taux d'accouchements par voie basse est passé de 54,8% (1 083 331/1 978 380) à 52,9% (1 091 958/2 064 507), ce qui correspond à une diminution relative du taux de 3,5%. Le taux d'accouchements par césarienne est passé de 43,9% (869 018/1 978 380) à 45,5% (940 206/2 064 507), ce qui correspond à une augmentation relative du taux de 3,7%. Le changement le plus important concernant les taux d'accouchements a été constaté dans les hôpitaux du secteur privé. Depuis 2014, les taux d'accouchements par césarienne ont légèrement diminué dans tous les secteurs, mais demeurent élevés (45,5%). Des politiques et des interventions appropriées sont nécessaires pour réduire le taux d'accouchements par césarienne aux Mexique, en particulier dans les hôpitaux de secteur privé.

## Resumen

Las tasas de parto por cesárea en México están entre las más altas del mundo. Dada la creciente concienciación pública y profesional sobre este problema y las directrices nacionales actualizadas de 2014 para reducir la frecuencia de los partos por cesárea, se analizaron las tendencias de los partos por cesárea según el tipo de establecimiento en México entre 2008 y 2017. Se obtuvieron datos de los certificados de nacimiento de la Dirección General de Información Sanitaria de México y se agrupó el número total de partos vaginales y por cesárea en cinco categorías de establecimientos: hospitales del ministerio de salud pública, hospitales privados, hospitales gubernamentales para asegurados por empleo, hospitales militares y otras instalaciones. Se calcularon los índices de partos para cada categoría a nivel nacional y según cada estado. De media, 2 114 630 (intervalo de confianza, IC, del 95 %: 2 061 487–2 167 773) nacimientos vivos se produjeron a nivel nacional al año entre 2008 y 2017. De estos nacimientos, el 53,5 % (1 130 570; IC del 95 %: 1 108 068–1 153 072) fueron partos vaginales y el 45,3 % (957 105; IC del 95 %: 922 936–991 274) fueron partos por cesárea, con poca variación a lo largo del tiempo. Durante el periodo de estudio, el número de nacidos vivos aumentó un 4,4 % (de 1 978 380 a 2 064 507). La tasa de partos vaginales disminuyó del 54,8 % (1 083 331/1 978 380) al 52,9 % (1 091 958/2 064 507), lo que supone una disminución porcentual relativa de la tasa del 3,5 %. La tasa de partos por cesárea aumentó del 43,9 % (869 018/1 978 380) al 45,5 % (940 206/2 064 507), lo que representa un aumento porcentual relativo de la tasa del 3,7 %. El mayor cambio en las tasas de partos se produjo en los hospitales del sector privado. Desde 2014, las tasas de parto por cesárea se han reducido ligeramente en todos los sectores, pero siguen siendo elevadas (45,5 %). Se necesitan políticas con intervenciones apropiadas para reducir la tasa de partos por cesárea en México, especialmente en los hospitales del sector privado.

## ملخص

تعد معدلات الولادة القيصرية في المكسيك واحدة من أعلى المعدلات على مستوى العالم. في ظل ارتفاع الوعي لدى العامة والمهنيين بهذه المشكلة وبالمبادئ التوجيهية الوطنية التي تم تعديلها عام 2014 للحد من وتيرة الولادة القيصرية، فقد قمنا بتحليل الاتجاهات في الولادة القيصرية حسب نوع المنشأة في المكسيك من عام 2008 إلى عام 2017. وحصلنا على بيانات شهادات الميلاد من الإدارة المكسيكية العامة للمعلومات الصحية، كما قمنا بتقسيم العدد الإجمالي من الولادات الطبيعية والقيصرية إلى خمس فئات من المرافق: مستشفيات وزارة الصحة؛ والمستشفيات الخاصة؛ ومستشفيات التأمين ذات فرق العمل الحكومية؛ والمستشفيات العسكرية، وغيرها من المرافق. تم حساب معدلات الولادة لكل فئة على المستوى الوطني ولكل ولاية. بمعدل متوسط، 2114630 (فاصل الثقة 95%: 2061487 إلى 2167773) حالة مواليد أحياء حدثت على المستوى الوطني كل عام ما بين عامي 2008 و2017. ونسبة 53.5% من هذه الولادات (1130570؛ فاصل الثقة 95%: 1108068 إلى 1153072) كانت ولادات طبيعية، ونسبة 45.3% منها (957105؛ فاصل الثقة 95%: 922936 إلى 991274) كانت ولادات قصية، فضلاً عن قليل من التنوع بمرور الوقت. وخلال فترة الدراسة، زاد عدد المواليد الأحياء بنسبة 4.4% (من 1978380 إلى 2064507). انخفض معدل الولادة الطبيعية من 54.8% (1083331/1978380) إلى 52.9% (1091958/2064507)، مما أدى بدوره إلى انخفاض نسبي في النسبة المئوية بمعدل 3.5%. كما انخفض معدل الولادة القيصرية من 43.9% (869018/1978380) إلى 45.5% (940206/2064507)، مما أدى بدوره إلى ارتفاع نسبي في النسبة المئوية بمعدل 3.7%. وكان أكبر تغيير في معدلات الولادة في مستشفيات القطاع الخاص. منذ عام 2014، تعرضت معدلات الولادة القيصرية للانخفاض بشكل طفيف في جميع القطاعات، لكنها لا تزال مرتفعة عند مستوى 45.5%. هناك حاجة إلى سياسات تنطوي على تدخلات مناسبة، وذلك للحد من معدل الولادة القيصرية في المكسيك، وخاصة في مستشفيات القطاع الخاص.

## 摘要

墨西哥剖腹产分娩率居世界首位。鉴于公众和专业人员对此问题的日益重视，以及修订后的 2014 年版国家指南旨在减少剖腹产手术发生率，我们按照机构类型对墨西哥 2008 年至 2017 年的剖腹产手术趋势进行了分析。我们从墨西哥卫生信息总局 (Mexican General Directorate for Health Information) 处获得了出生证明的数据，并将阴道分娩和剖腹产分娩的总数按照机构类型分为五类：卫生部下辖医院；私立医院；政府就业保险医院；军队医院；以及其它医院。计算全国以及各州各类型机构的分娩率。2008 年至 2017 年期间，全国年均活产儿为 2 114 630（95％ 置信区间，CI：2 061 487-2 167 773）。在这些活产儿中，53.5％（1 130 570；95％ 置信区间，CI：1 108 068-1 153 072）为阴道分娩，45.3％（957 105；95％ CI：922 936-991 274）为剖腹产分娩，数据随时间变化极小。研究期间，活产儿数量增加了 4.4％（从 1 978 380 增加至 2 064 507 ）。阴道分娩率从 54.8％（1 083 331/1 978 380）降至 52.9％（1 091 958/2 064 507），相对百分比下降 3.5％。剖腹产率从 43.9％（869 018/1 978 380）上升至 45.5％（940 206/2 064 507），相对百分比上升 3.7％。分娩率上的最大变化出现在私营医院。自 2014 年以来，虽然所有机构的剖腹产分娩率略有下降，但仍高达 45.5％。需施行政策，采取适当干预措施降低墨西哥（尤其是私营医院）的剖腹产分娩率**。**

## Резюме

Мексика характеризуется одним из самых высоких показателей родоразрешения с помощью кесарева сечения в мире. С учетом признания этой проблемы специалистами и общественностью и с учетом обновленных национальных рекомендаций от 2014 года, направленных на снижение количества кесаревых сечений, авторы статьи анализируют тенденции в использовании кесарева сечения в зависимости от типа медицинского учреждения в Мексике в период с 2008 по 2017 год. От Главного управления здравоохранения Мексики были получены данные свидетельств о рождении, которые затем были сгруппированы с разделением общего количества вагинальных родов и родов с использованием кесарева сечения в пять категорий по типу медицинского учреждения: больницы Министерства здравоохранения, частные больницы, государственные больницы, предоставляющие услуги по страховке для работающих, военные госпитали и прочие медицинские учреждения. Были рассчитаны показатели родов для каждой категории в национальном масштабе и отдельно по штатам. В среднем ежегодно в период между 2008 и 2017 годами регистрировалось 2 114 630 живорожденных младенцев (95%-й ДИ: 2 061 487–2 167 773). Из них 53,5% (1 130 570; 95%-й ДИ: 1 108 068–1 153 072) приходилось на вагинальные роды и 45,3% (957 105; 95%-й ДИ: 922 936–991 274) родились с помощью кесарева сечения; вариации по годам были незначительными. В период исследования количество живорожденных выросло на 4,4% (с 1 978 380 до 2 064 507). Количество вагинальных родов снизилось с 54,8% (1 083 331 из 1 978 380) до 52,9% (1 091 958 из 2 064 507), что дает относительное процентное уменьшение на 3,5%. Частота кесарева сечения выросла с 43,9% (869 018 из 1 978 380) до 45,5% (940 206 из 2 064 507), что дает относительное процентное увеличение на 3,7%. Максимальное изменение показателей отмечалось в частных больницах. С 2014 года частота кесарева сечения слегка снизилась во всех учреждениях, но остается по-прежнему высокой (на уровне 45,5%). Необходимо разработать стратегию и соответствующие меры, которые позволили бы снизить частоту использования кесарева сечения в Мексике, особенно в больницах частного сектора.

## Introduction

Caesarean delivery is a vital procedure to reduce maternal and neonatal mortality.^1^ However, in some middle- and high-income settings, caesarean deliveries have increased sharply.^1^ Although no clear optimal rate has been established as a threshold, a caesarean delivery rate of up to 19 per 100 live births is associated with the lowest rates of maternal and neonatal mortality at a population level.^2^

Caesarean delivery rates in Mexico, a country with the second largest economy in Latin America^3^ and with a population of nearly 120 million^4^, are among the highest in the world. For example, the national rate of caesarean delivery in first-time mothers was 48.7% (292 445/600 124) in 2014, with higher rates in private facilities than non-private facilities, regardless of type of insurance coverage.^5^These rates are of concern because high rates of caesarean delivery can result in harmful consequences for both the mother and baby.^6^^,^^7^ The government^8^^,^^9^ and the public^9^ have been aware of this problem since the early 2000s. More recently, two newspaper articles^10^^,^^11^ described several cases of unnecessary caesarean delivery, those performed without medical indication,^12^^,^^13^ and subsequent morbidity. These cases indicate that Mexico has a high burden of harmful overtreatment during childbirth.

The health ministry reported a considerable increase in unnecessary caesarean deliveries in the public and private sectors in 2002^9^ and provided guidelines for indications to perform caesarean deliveries and strategies to reduce their frequency.^9^ In 2014, the ministry published updated guidelines to further reduce caesarean deliveries.^14^ In the same year, the Mexican Social Security Institute (IMMS), a government affiliated employment-based insurance network, also published clinical practice guidelines to reduce the frequency of caesarean deliveries.^8^ The clinical practice guidelines were widely disseminated and endorsed by other government-affiliated employment-based insurance networks (Institute for Social Security and Services for State Workers, and PEMEX – Mexican Petroleum), the health ministry, military sectors, and academia. Mexico’s national policy on caesarean delivery was again updated in 2016.^15^

Given the heightened public and professional awareness of the high rate of caesarean delivery and the 2014 updated national guidelines to reduce the frequency of caesarean deliveries,^8^^,^^14^ we analysed the trends in caesarean delivery in health-care facilities in Mexico from 2008 to 2017 to assess their impact on caesarean delivery.

## Methods

### Study design and data source

We conducted an ecological analysis of data from publicly available birth certificates from the General Directorate for Health Information of the Mexican health ministry for the period 2008 to 2017.^16^ This data set includes all annual live births with a birth certificate in Mexico and provides demographic and clinical information on both mothers and their newborns.

### Variables

We extracted data on the following variables for each of the 32 Mexican states and overall: total live births; mode of delivery (vaginal delivery, caesarean delivery, forceps-assisted vaginal delivery; complicated delivery such as vaginal breech delivery, other modes of delivery and unspecified mode of delivery); and the organizations funding the facility where delivery occurred. The health-care facilities were the health ministry; the Mexican Social Security Institute (IMSS), a tax-funded government institution that provides employment-based insurance and health services to its beneficiaries and retirees; IMSS-Oportunidades, a government programme that extends social and health services to rural and urban, marginalized and indigenous populations; the Institute for Social Security and Services for State Workers, which provides health-care coverage for government employees; PEMEX, which provides health-care coverage for its employees, retirees and their families; the Office for National Defence, which provides health-care coverage for its employees, retirees and family members of individuals affiliated with Mexico’s army and air force; the Office for the Navy which provides health-care coverage for its employees, retirees and family members of individuals affiliated with the Mexican Navy; and other public and private facilities, roadside delivery (on the way to a health-care facility), home delivery, other, and unspecified.

The outcome variables were the total number of vaginal, caesarean and other deliveries, which were categorized into five types of facility: (i) health-ministry hospitals, (ii) private hospitals, (iii) government employment-based insurance hospitals (Social Security Institute, IMSS-Oportunidades, Institute for Social Security and Services for State Workers, and PEMEX), (iv) military hospitals (Office for National Defence and Office for the Navy), and (v) other facilities. Delivery rates were calculated for each category of health facility and overall, nationally and by state. We calculated the difference in the rates of vaginal and caesarean delivery between 2008 and 2017 and present this relative change in rate as a percentage of the 2008 rate.

### Statistical analysis

We performed multivariable logistic regression with the year as a continuous covariate to test for trends and determine if there were statistically significant differences (*P* < 0.05) between rates of caesarean delivery within each type of facility over time, using the health-ministry facilities as the reference category. Statistical analyses were performed using SAS 9.4 (SAS Institute, Cary, United States of America). 

## Findings

### National and state 

**Fig. 1:**
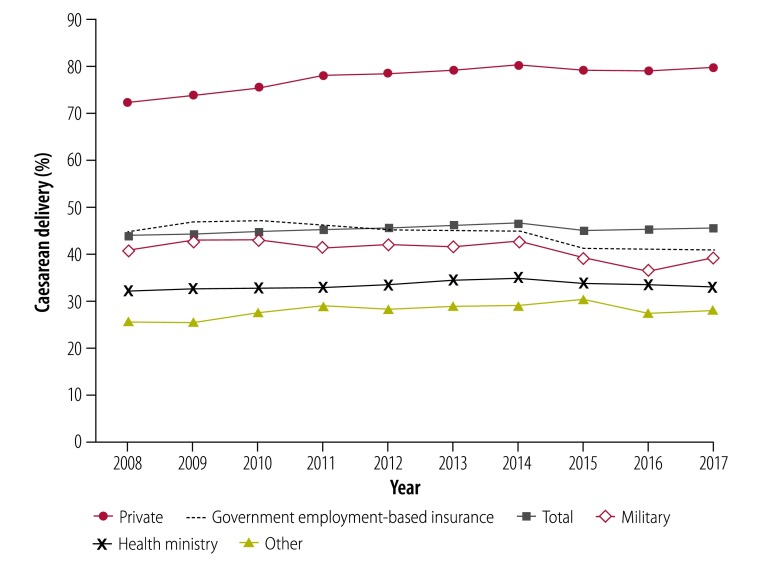
Rates of caesarean delivery by sector, Mexico, 2008–2017

Note: The values are percentages of total live births per year.

There were on average 2 114 630 (95% confidence interval, CI: 2 061 487–2 167 773) live births a year nationally between 2008 and 2017, of which 1 130 570 (95% CI: 1 108 068–1 153 072) were vaginal deliveries and 957 105 (95% CI: 922 936–991 274) were caesarean deliveries. National rates for vaginal and caesarean delivery were 53.5% and 45.3%, respectively, with little variation over time (Fig. 1). The number of overall live births increased by 4.4% (from 1 978 380 to 2 064 507) during this 10-year period. The rate of vaginal delivery decreased by 1.9 percentage points (from 54.8% [1 083 331/1 978 380] to 52.9% [1 091 958/2 064 507]; Table 1), giving a relative percentage decrease in the vaginal delivery rate of 3.5%. The rate of caesarean delivery increased by 1.6 percentage points (from 43.9% [869 018/1978380] to 45.5% [940 206/2 064 507]), giving a relative percentage increase in the caesarean delivery rate of 3.7% (Table 1). 

**Table 1:** Live births by mode of delivery, Mexico, 2008–2017

Year	No. of total live births	Vaginal deliveries, no. (%)	Caesarean deliveries, no. (%)	Other deliveries, no. (%)^a^
2008	1 978 380	1 083 331 (54.8)	869 018 (43.9)	26 031 (3.0)
2009	2 058 708	1 119 422 (54.4)	913 545 (44.4)	25 741 (2.8)
2010	2 073 111	1 120 123 (54.0)	928 299 (44.8)	24 689 (2.7)
2011	2 167 060	1 163 844 (53.7)	978 144 (45.1)	25 072 (2.6)
2012	2 206 692	1 177 244 (53.3)	1 005 897 (45.6)	23 551 (2.3)
2013	2 195 073	1 156 978 (52.7)	1 014 517 (46.2)	23 578 (2.3)
2014	2 177 319	1 140 835 (52.4)	1 014 336 (46.6)	22 148 (2.2)
2015	2 145 199	1 146 219 (53.4)	966 607 (45.1)	32 373 (3.3)
2016	2 080 253	1 105 745 (53.2)	940 479 (45.2)	34 029 (3.6)
2017	2 064 507	1 091 958 (52.9)	940 206 (45.5)	32 343 (3.4)

^a^ Other deliveries are forceps, complicated deliveries, other and unspecified.

**Fig. 2:**
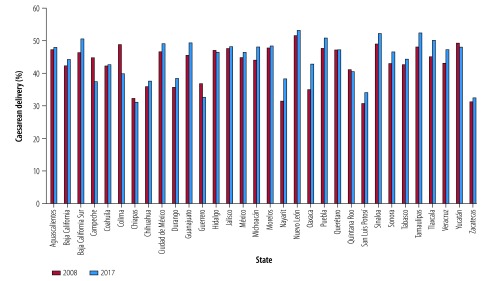
Rates of caesarean delivery, by state, Mexico, 2008 and 2017

Note: The values are percentages of total live births per year.

**Fig. 3:**
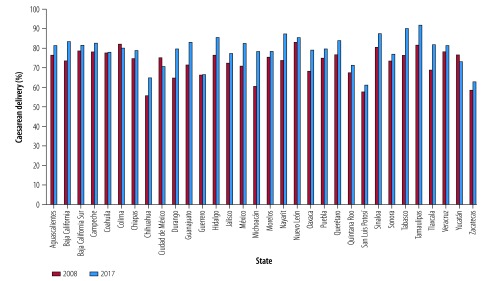
Rates of caesarean delivery in private facilities by state, Mexico, 2008 and 2017

Note: The values are percentages of total live births per year.

**Fig. 4:**
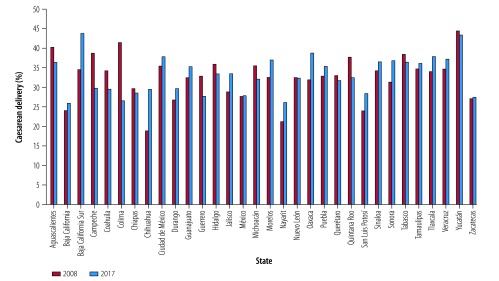
Rates of caesarean delivery, in health-ministry facilities by state, Mexico, 2008 and 2017

Note: The values are percentages of total live births per year.

**Fig. 5:**
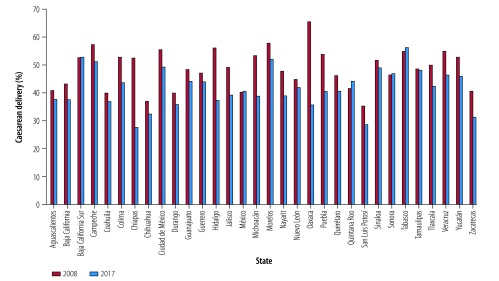
Rates of caesarean delivery in employment-based insurance facilities by state, Mexico, 2008 and 2017

Note: The values are percentages of total live births per year.

Data for 2008 show substantial variation in the overall rates of caesarean delivery by state, ranging from 31% in Nayarit, San Luis Potosi and Zacatecas to 51% in Nuevo Leon (Fig. 2). In the private sector, the variation was even greater than the overall rates, ranging from 56% in Chihuahua to 83% in Nuevo Leon (Fig. 3). In 2017, overall rates of caesarean delivery varied considerably by state, ranging from 31% in Chiapas to 53% in Nuevo Leon (Fig. 2). Again, in the private sector, the variation was even greater, ranging from 61% in San Luis Potosi to 92% in Tamaulipas (Fig. 3). Fig. 4 and Fig. 5 show the rates of caesarean delivery in health-ministry and employment-based insurance hospitals, respectively, in 2008 and 2017. 

The rates of vaginal and caesarean delivery by state are shown in Table 2. In most states (25 out of 32), the rate of caesarean delivery increased from 2008 to 2017, but seven states showed lower caesarean delivery rates. This decrease was particularly noteworthy in Colima (49% to 40%) and Campeche (45% to 37%; Fig. 2).

**Table 2:** Live births by state and mode of delivery, Mexico, 2008 and 2017

State	No. of total live births			Vaginal deliveries, no. (%)			Caesarean deliveries, no. (%)			Other deliveries, no. (%)^a^	
	2008	2017		2008	2017		2008	2017		2008	2017
Aguascalientes	26 741	29 045		13 428 (50.2)	14 816 (51.0)		12 645 (47.3)	13 932 (48.0)		668 (2.5)	297 (1.0)
Baja California	46 713	53 086		26 522 (56.8)	29 334 (55.3)		19 772 (42.3)	23 495 (44.3)		419 (0.9)	257 (0.5)
Baja California Sur	11 180	11 891		5 893 (52.7)	5 757 (48.4)		5 182 (46.4)	6 010 (50.5)		105 (0.9)	124 (1.0)
Campeche	13 369	14 098		7 317 (54.7)	8 738 (62.0)		5 996 (44.9)	5 283 (37.5)		56 (0.4)	77 (0.5)
Chiapas	64 167	90 897		43 119 (67.2)	60 312 (66.4)		20 798 (32.4)	28 263 (31.1)		250 (0.4)	2322 (2.6)
Chihuahua	54 167	61 534		34 125 (63.0)	37 287 (60.6)		19 516 (36.0)	23 165 (37.6)		526 (1.0)	1082 (1.8)
Ciudad de México	142 110	132 363		73 778 (51.9)	65 025 (49.1)		66 274 (46.6)	64 932 (49.1)		2058 (1.4)	2406 (1.8)
Coahuila	55 121	57 274		30 959 (56.2)	31 809 (55.5)		23 294 (42.3)	24 482 (42.7)		868 (1.6)	983 (1.7)
Colima	12 731	12 676		6 460 (50.7)	7 522 (59.3)		6 217 (48.8)	5 058 (39.9)		54 (0.4)	96 (0.8)
Durango	29 036	32 538		18 420 (63.4)	19 723 (60.6)		10 354 (35.7)	12 495 (38.4)		262 (0.9)	320 (1.0)
Guanajuato	117 299	116 367		61 760 (52.7)	56 489 (48.5)		53 515 (45.6)	57 479 (49.4)		2024 (1.7)	2399 (2.1)
Guerrero	45 070	60 081		28 310 (62.8)	39 827 (66.3)		16 636 (36.9)	19 646 (32.7)		124 (0.3)	608 (1.0)
Hidalgo	47 702	46 773		25 131 (52.7)	24 281 (51.9)		22 459 (47.1)	21 782 (46.6)		112 (0.2)	710 (1.5)
Jalisco	134 579	140 725		68 160 (50.6)	69 351 (49.3)		64 075 (47.6)	67 948 (48.3)		2344 (1.7)	3426 (2.4)
México	290 337	258 101		158 385 (54.6)	136 791 (53.0)		130 463 (44.9)	119 844 (46.4)		1489 (0.5)	1466 (0.6)
Michoacán	82 883	86 942		45 784 (55.2)	44 873 (51.6)		36 548 (44.1)	41 930 (48.2)		551 (0.7)	139 (0.2)
Morelos	31 860	31 550		16 565 (52.0)	15 741 (49.9)		15 234 (47.8)	15 277 (48.4)		61 (0.2)	532 (1.7)
Nayarit	18 969	17 979		12 920 (68.1)	10 895 (60.6)		5 969 (31.5)	6 892 (38.3)		80 (0.4)	192 (1.1)
Nuevo León	76 278	92 642		27 919 (36.6)	36 027 (38.9)		39 261 (51.5)	49 234 (53.1)		9098 (11.9)	7381 (8.0)
Oaxaca	41 869	69 747		27 071 (64.7)	39 204 (56.2)		14 680 (35.1)	29 890 (42.9)		118 (0.3)	653 (0.9)
Puebla	111 821	125 336		58 248 (52.1)	60 757 (48.5)		53 387 (47.7)	63 798 (50.9)		186 (0.2)	781 (0.6)
Querétaro	40 195	41 233		20 760 (51.6)	21 273 (51.6)		18 970 (47.2)	19 488 (47.3)		465 (1.2)	472 (1.1)
Quintana Roo	23 576	27 915		13 722 (58.2)	16 037 (57.4)		9 715 (41.2)	11 317 (40.5)		139 (0.6)	561 (2.0)
San Luis Potosí	49 125	48 007		33 108 (67.4)	30 665 (63.9)		15 101 (30.7)	16 397 (34.2)		916 (1.9)	945 (2.0)
Sinaloa	50 858	50 872		25 761 (50.7)	24 237 (47.6)		24 943 (49.0)	26 502 (52.1)		154 (0.3)	133 (0.3)
Sonora	49 327	44 958		27 848 (56.5)	23 685 (52.7)		21 269 (43.1)	20 963 (46.6)		210 (0.4)	310 (0.7)
Tabasco	50 247	47 877		28 381 (56.5)	26 354 (55.0)		21 441 (42.7)	21 274 (44.4)		425 (0.8)	249 (0.5)
Tamaulipas	68 054	57 602		34 065 (50.1)	26 487 (46.0)		32 787 (48.2)	30 192 (52.4)		1202 (1.8)	923 (1.6)
Tlaxcala	23 208	23 896		12 681 (54.6)	11 559 (48.4)		10 480 (45.2)	12 007 (50.2)		47 (0.2)	330 (1.4)
Veracruz	106 621	114 921		60 279 (56.5)	59 263 (51.)		45 969 (43.1)	54 405 (47.3)		373 (0.3)	1253 (1.1)
Yucatán	35 070	35 573		17 591 (50.2)	18 182 (51.1)		17 274 (49.3)	17 076 (48.0)		205 (0.6)	315 (0.9)
Zacatecas	28 097	30 004		18 861 (67.1)	19 654 (65.5)		8 794 (31.3)	9 750 (32.5)		442 (1.6)	600 (2.0)

^a^ Other deliveries are forceps, problematic deliveries, other and unspecified.

### Health-ministry facilities

In health-ministry facilities, there were 1 006 514 (95% CI: 968 497–1 044 531) deliveries a year on average between 2008 and 2017, of which 660 235 (95% CI: 637 926–682 544) were vaginal deliveries and 335 771 (95% CI: 318 784–352 759) were caesarean deliveries. In the public sector, 65.6% of births were vaginal delivery and 33.4% were caesarean delivery, with little variation over time (Fig. 1). The number of live births in health-ministry facilities increased by 7.0% (from 891 023 to 953 825) during the 10-year period. The rate of vaginal delivery decreased by 0.7 percentage points (from 66.6% to 65.9%; Table 3), giving a relative percentage decrease in the vaginal delivery rate of 1.0%. The caesarean delivery rate increased by 0.8 percentage points (from 32.2% to 33.0%; Table 3) giving a relative percentage increase in the caesarean delivery rate of 2.7%. Caesarean delivery in health-ministry facilities has gradually decreased since 2014, from 34.9% to 33.0% in 2017 (Table 3).

**Table 3:** Mode of delivery by facility, Mexico, 2008–2017

Year	No. of total live births	Vaginal deliveries, no. (%)	Caesarean deliveries, no. (%)	Other deliveries, no. (%)^a^
**Health-ministry facilities**				
2008	891 023	593 563 (66.6)	286 540 (32.2)	10 920 (1.2)
2009	988 826	655 255 (66.3)	322 576 (32.6)	10 995 (1.1)
2010	1 018 289	673 967 (66.2)	333 062 (32.7)	11 260 (1.1)
2011	1 051 779	695 015 (66.1)	345 762 (32.9)	11 002 (1.0)
2012	1 060 571	695 124 (65.5)	355 007 (33.5)	10 440 (1.0)
2013	1 044 013	674 955 (64.7)	359 225 (34.4)	9 833 (0.9)
2014	1 045 159	670 942 (64.2)	364 984 (34.9)	9 233 (0.9)
2015	1 027 982	670 380 (65.2)	346 761 (33.7)	10 841 (1.1)
2016	983 672	644 115 (65.5)	328 642 (33.4)	10 915 (1.1)
2017	953 825	629 035 (65.9)	315 153 (33.0)	9 637 (1.0)
**Private facilities**				
2008	420 866	114 193 (27.1)	304 432 (72.3)	2241 (0.5)
2009	409 083	104 571 (25.6)	302 707 (74.0)	1805 (0.4)
2010	414 353	99 743 (24.1)	313 140 (75.6)	1470 (0.4)
2011	424 570	91 604 (21.6)	331 369 (78.0)	1597 (0.4)
2012	446 416	94 028 (21.1)	350 947 (78.6)	1441 (0.3)
2013	442 888	90 630 (20.5)	350 879 (79.2)	1379 (0.3)
2014	439 936	85 546 (19.4)	352 994 (80.2)	1396 (0.3)
2015	444 782	87 404 (19.7)	351 880 (79.1)	5498 (1.2)
2016	456 419	87 611 (19.2)	361 136 (79.1)	7672 (1.7)
2017	463 826	85 288 (18.4)	370 049 (79.8)	8489 (1.8)
**Employment-based insurance facilities**				
2008	551 250	292 964 (53.1)	246 417 (44.7)	11 869 (2.2)
2009	552 502	282 027 (51.0)	258 359 (46.8)	12 116 (2.2)
2010	534 147	272 279 (51.0)	250 800 (47.0)	11 068 (2.1)
2011	579 401	300 690 (51.9)	267 248 (46.1)	11 463 (2.0)
2012	592 562	314 097 (53.0)	267 779 (45.2)	10 686 (1.8)
2013	601 196	318 228 (52.9)	271 818 (45.2)	11 150 (1.9)
2014	591 372	315 742 (53.4)	265 250 (44.9)	10 380 (1.8)
2015	577 528	325 062 (56.3)	238 219 (41.2)	14 247 (2.5)
2016	558 101	317 081 (56.8)	227 148 (40.7)	13 872 (2.5)
2017	566 966	322 772 (56.9)	231 458 (40.8)	12 736 (2.2)
**Military facilities**				
2008	13 924	8170 (58.7)	5687 (40.8)	67 (0.5)
2009	13 072	7426 (56.8)	5616 (43.0)	30 (0.2)
2010	12 911	7307 (56.6)	5571 (43.1)	33 (0.3)
2011	13 317	7764 (58.3)	5492 (41.2)	61 (0.5)
2012	13 878	7988 (57.6)	5840 (42.1)	50 (0.4)
2013	13 677	7922 (57.9)	5700 (41.7)	55 (0.4)
2014	13 363	7611 (57.0)	5706 (42.7)	46 (0.3)
2015	12 359	7398 (59.9)	4849 (39.2)	112 (0.9)
2016	11 618	7247 (62.4)	4221 (36.3)	150 (1.3)
2017	10 628	6383 (60.1)	4168 (39.2)	77 (0.7)
**Other facilities**				
2008	101 317	74 441 (73.5)	25 942 (25.6)	934 (0.9)
2009	95 225	70 143 (73.7)	24 287 (25.5)	795 (0.8)
2010	93 411	66 827 (71.5)	25 726 (27.5)	858 (0.9)
2011	97 993	68 771 (70.2)	28 273 (28.9)	949 (1.0)
2012	93 265	66 007 (70.8)	26 324 (28.2)	934 (1.0)
2013	93 299	65 243 (69.9)	26 895 (28.8)	1161 (1.2)
2014	87 489	60 994 (69.7)	25 402 (29.0)	1093 (1.2)
2015	82 548	55 975 (67.8)	24 898 (30.2)	1675 (2.0)
2016	70 443	49 691 (70.5)	19 332 (27.4)	1420 (2.0)
2017	69 262	48 480 (70.0)	19 378 (28.0)	1404 (2.0)

^a^ Other deliveries are forceps, problematic deliveries, other and unspecified.

### Private facilities

In the private sector, there were 436 314 (95% CI: 423 272–449 356) deliveries a year on average between 2008 and 2017, of which 94 062 (95% CI: 87 312–100 812) were vaginal deliveries, and 338 953 (95% CI: 321 531–356 376) were caesarean deliveries. In private facilities, 21.6% of births were vaginal delivery and 77.7% were caesarean delivery, with little variation over time (Fig. 1). The number of live births increased by 10.2% (from 420 866 to 463 826) during the 10-year period. The rate of vaginal delivery decreased by 8.7 percentage points (from 27.1% to 18.4%; Table 3), giving a relative percentage decrease in the rate of vaginal delivery of 32.2%. The caesarean delivery rate increased by 7.5 percentage points (from 72.3% to 79.8%; Table 3) giving a relative percentage decrease in the caesarean delivery rate of 10.3%. The change in the rate of caesarean delivery over this 10-year period in the private sector was statistically significant compared with the change in rate in the public sector (*P* < 0.001). The rate of caesarean delivery in private facilities was 80.2% in 2014 and showed a slight decrease in 2015 and 2016 to 79.1%, but the rate increased again in 2017 to 79.8%.

### Employment insurance facilities 

In government employment-based insurance facilities, there were 570 503 (95% CI: 555 094–585 911) live births a year on average between 2008 and 2017, of which 306 094 (95% CI: 293 061–319 127) were vaginal deliveries, and 252 450 (95% CI: 240 889–264 010) were caesarean deliveries. In these facilities, 53.7% of live births were vaginal delivery and 44.3% were caesarean delivery, with little variation over time (Fig. 1). The number of live births increased by 2.9% (from 551 250 to 566 966) during the 10-year period. The rate of vaginal delivery increased by 3.8 percentage points (from 53.1% to 56.9%; Table 3), giving a relative percentage decrease in the vaginal delivery rate of 7.1%. The rate of caesarean delivery decreased by 3.9 percentage points (from 44.7% to 40.8%; Table 3), giving a relative percentage decrease in the caesarean delivery rate of 8.7%.

The change in the rate of caesarean delivery over this 10-year period in government employment-based insurance facilities was statistically significant compared with the change in rate in the public sector (*P* < 0.001).

Caesarean delivery rates in government employment-based insurance facilities decreased from 44.9% in 2014, when the clinical practice guidelines were published, to 41.2% in 2015 and thereafter plateaued.

**Fig. 6:**
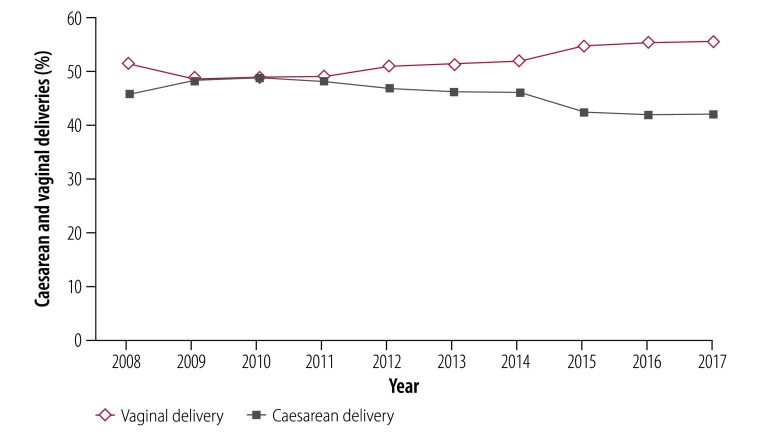
Rates of caesarean and vaginal delivery in facilities of the Social Security Institute, Mexico, 2008–2017

Notes: The values are percentages of total live births per year. The Social Security Institute is financed by employment-based insurance.

In a subanalysis of facilities of the Mexican Social Security Institute, caesarean delivery decreased gradually from 46.2% (210 864/456 826) in 2008 to 42.5% (183 700/431 775) in 2015, with a subsequent decrease to 41.9% (177 965/424 454) in 2017 (Fig. 6). The rate of vaginal delivery increased from 51.4% (234 659/456 826) to 55.6% (235 827/424 454) between 2008 and 2017, a difference of 4.2 percentage points, giving a relative percentage increase in the vaginal delivery rate of 8.2%. The caesarean delivery rate decreased from 46.2% (210 864/456 826) to 41.9% (177 965/424 454), a difference of 4.3 percentage points, giving a relative percentage decrease in the caesarean delivery rate of 9.2%. After the introduction of the clinical practice guidelines in 2014, caesarean delivery rates decreased from 46.0% (206 787/449 059) in 2014 to 41.9% (177 965/424 454) in 2017.

### Military facilities

In military facilities, there were 12 875 (95% CI: 12 116–13 633) deliveries a year on average between 2008 and 2017, of which 7522 (95% CI: 7159–7884) were vaginal deliveries and 5285 (95% CI: 4831–5739) were caesarean deliveries. In military facilities, 58.4% of births were vaginal delivery and 41.0% were caesarean delivery, with little variation over time (Fig. 1). The number of live births in military facilities decreased by 23.7% (from 13 924 to 10 628) during the 10-year period. The rate of vaginal delivery increased by 1.4 percentage points (from 58.7% to 60.1%; Table 3) giving a relative percentage increase in the rate of vaginal delivery of 2.4%. The rate of caesarean delivery decreased by 1.6 percentage points (from 40.8% to 39.2%; Table 3), giving a relative percentage increase in the rate of caesarean delivery of 4.0%. There was no statistically significant difference in the change in rate of caesarean delivery in military facilities over this 10-year period compared with the change in rate in the public sector (*P* = 0.28).

More recently, military facilities showed a decrease in rates of caesarean delivery, from 42.7% in 2014 to 36.3% in 2016, but they rose again in 2017 to 39.2%.

### Other facilities

In other facilities, there were 88 425 (95% CI: 80 509–96 341) deliveries a year on average between 2008 and 2017, of which 62 657 (95% CI: 56 420–68 894) were vaginal deliveries, and 24 646 (95% CI: 22 505–26 787) were caesarean deliveries. In other facilities, the rate of vaginal delivery was 70.9% and the rate of caesarean delivery was 27.9%, with little variation over time (Fig. 1). There was a 31.6% decrease (from 101 317 to 69 262) in the total number of live births during this 10-year period. The rate of vaginal delivery decreased by 3.5 percentage points (from 73.5% to 70.0%; Table 3), giving a relative percentage decrease in the rate of vaginal delivery of 4.7%. The rate of caesarean delivery increased by 2.4 percentage points (from 25.6% to 28.0%; Table 3), giving a relative percentage increase in caesarean delivery of 9.3%. There was no statistically significant difference in the change in the rate of caesarean delivery in other facilities over this 10-year period compared with the change in rate in the public sector (*P* = 0.39).

## Discussion

Caesarean delivery rates are still alarmingly high in Mexico and increased between 2008 and 2017, with the biggest increase in private hospitals. These trends were statistically significant in the private and the employment-based insurance facilities compared with health-ministry facilities. However, in 2015 and 2016, after the 2014 clinical practice guidelines were published, rates of caesarean delivery decreased slightly in all types of facility, although they rose again in 2017 in all but health-ministry facilities. These findings illustrate the difficulty in implementing and sustaining change across a mulitsectoral health-care system. 

The 2014 clinical practice guidelines of the Social Security Institute aimed to reduce the number of unnecessary caesarean deliveries.^8^ Our subanalysis of trends in caesarean deliveries in Social Security Institute facilities showed an overall decrease in the rate of caesarean delivery during the 10-year period, with the greatest decrease after the introduction of the clinical practice guidelines. The Social Security Institute monitored the effect of the guidelines through an electronic verification registry score system which assigns points (1, 0, not available) for each recommendation followed. Recommendations are categorized as: strategies to reduce caesarean deliveries, diagnostic tests, labour management, and technical criteria for referral. For example, adherence to the clinical guidelines was 60% in one hospital using the score system of the Social Security Institute (personal communication, Dr Maria Antonia Basavilvazo-Rodriguez, 2019), which is well below the goal.

Lack of compliance with the recommendations on caesarean delivery could be associated with factors at different levels: the health system and facilities, health professionals, and patients and their communities. Regarding health system and facility factors, the health-care infrastructure varies widely by sector and state, including in human resources, labour rooms and quality committees to evaluate caesarean deliveries. Health professionals may resist following updated clinical guidelines because of habit and perverse financial incentives (e.g. they get paid more for caesarean deliveries than vaginal deliveries). For women and the community, health professionals need to provide clear and accurate information about the benefits of vaginal delivery, including the options for pain control, and for caesarean delivery when clinically indicated. Reinforcing the dissemination and implementation of the clinical guidelines and regulating financial incentives are both needed to ensure health professionals follow the national policy on caesarean delivery.

Nationally, one could argue that the national policy had a positive effect because caesarean delivery rates showed a slight, but promising decrease in 2015. Unfortunately, after 2015, the overall rates have gradually increased, but have not reached the 2014 level. States showed variation in caesarean delivery rates; states with more resources had higher overall caesarean delivery rates than those with fewer resources, on average. In all states, the lowest caesarean delivery rates were in health-ministry hospitals (except Oaxaca in 2017 where the lowest rate was in government employment-based insurance facilities) and the highest rates were in private facilities in both 2008 and 2017. States where caesarean delivery decreased or increased considerably over the 10-year period should be further investigated to identify strategies that work and do not work so that successful interventions can be tailored and applied in other states.

The large difference between caesarean delivery rates in the private sector compared with other sectors is a cause for concern. Factors that may explain this difference include perverse economic incentives which exist at all levels of the health-care system: at the health system level (i.e. insurance coverage for caesarean delivery only), facility level (i.e. for-profit hospitals),^17^ and the physician level (i.e. induced demand for caesarean delivery,^18^ increased income through higher reimbursement for caesarean delivery than vaginal delivery). In addition, patients’ perceptions and preferences (e.g. fear of pain during delivery)^19^ can affect caesarean delivery rates. In fact, while policies on caesarean delivery provide useful guidance aimed at reducing the number of unnecessary caesarean deliveries based on clinical evidence, the technical guideline also highlights two points of concern: that some insurance policies only cover caesarean delivery and not vaginal delivery, and that women are requesting caesarean delivery rather than vaginal delivery to avoid pain, the slow progression of labour and perceived harm to their newborns with vaginal delivery. 

The policies and guidelines are unlikely to reverse the trend in caesarean delivery unless they are part of a multilevel, multistakeholder approach that has continuing support.^8^ A multipronged approach tailored to the local context that includes clinical and non-clinical health-care interventions has been proposed as a means to optimize the use of caesarean delivery.^12^ For example, a mandatory second opinion before a caesarean delivery can be performed has been proposed.^20^ Some suggested non-clinical interventions that are relevant to Mexico include: sharing appropriate evidence-based information on caesarean and vaginal delivery with women and their communities; creating financial arrangements that do not reward caesarean delivery and penalize vaginal delivery; and strengthening systems to provide trained staff and adequate pain relief in childbirth care.^12^^,^^21^

While higher socioeconomic status has been associated with an increase in caesarean delivery,^22^^,^^23^ vulnerable populations, such as indigenous groups, are also at risk of unnecessary caesarean delivery and should be monitored when assessing the effect of policies on caesarean delivery.^23^ Unfortunately, vulnerable populations who had access to health care through Mexico’s universal health-care insurance, Seguro Popular, might again be at risk, given current attempts to abolish it.^24^ Reversal of imperfect yet successful programmes such as Seguro Popular is likely to negatively affect efforts to reduce caesarean delivery in the public sector. If these programmes are reduced or abolished, the effect on maternal and neonatal health care, including on caesarean delivery, will require close monitoring and further research.

Mexico has a robust data collection system with several publicly-available data sets. However, improvements could be made in capturing relevant indicators of maternal and neonatal health, creating a system for quality assurance of data, and standardizing the definitions and classification of variables. Indications for caesarean delivery are poorly documented in both public and private sectors, which could be improved through audits and feedback.^13^ In addition, linking data sets, using unique record identifiers that protect the identity of individuals, is important, so that the clinical effects of high rates of caesarean delivery can be monitored over time, such as, hysterectomy during caesarean because of abnormal placentation.

Our study has two main limitations. First, we used publicly available data from birth certificates and births that occurred without a birth certificate were not included. Second, the analysis is based on live births and important information on maternal outcomes from stillbirths and abortions is not captured. Despite these limitations, the data set covers about 98% of Mexico’s population.^25^

## Conclusion

Reducing caesarean delivery rates in Mexico will require more than public awareness, guidelines and policies. First, an improved data collection and quality assurance system is necessary to better understand the consequences of high caesarean delivery rates over time. Second, increased oversight and regulation of private insurance companies is needed to reverse the perverse economic incentives that contribute to a very high caesarean delivery rate in the private sector. Finally, the medical and public health community must take an active role in educating the next generation of obstetricians and gynaecologists, the public and the insurance industry on the well documented benefits of vaginal delivery for both women and their newborns. Multilevel interventions, such as those available to improve quality of care for member countries of the Organization for Economic Co-operation and Development,^26^ are urgently needed to safely reduce the high rate of caesarean delivery in Mexico, particularly in private-sector hospitals.
